# Role of pyridoxine and oxidative stress in asthenozoospermia

**DOI:** 10.1016/j.heliyon.2024.e34799

**Published:** 2024-07-19

**Authors:** Roba Bdeir, Shefa’ Muneer Aljabali, Saleem Ali Banihani

**Affiliations:** a Department of Allied Health Sciences, Faculty of Nursing, Al-Balqa Applied University, As-salt, Jordan; b Department of Pharmaceutical Sciences, Faculty of Pharmacy, Jadara University, Irbid, Jordan; c Department of Medical Laboratory Sciences, Faculty of Allied Medical Sciences, Jadara University, Irbid, Jordan; d Department of Medical Laboratory Sciences, Jordan University of Science and Technology, Irbid, Jordan

**Keywords:** Seminal plasma, Vitamin B_6_, Glutathione, Total antioxidant capacity, Sperm parameters, Pyridoxine

## Abstract

**Purpose:**

Infertility is a worldwide concern, and recent research indicates that vitamin B_6_ deficiency may play a role in male infertility, primarily by inducing hyperhomocysteinemia and oxidative stress. These processes can have a detrimental effect on semen quality, ultimately affecting male fertility. Here, we aim to evaluate the biochemical status of pyridoxine (vitamin B_6_) in relation to total glutathione and total antioxidant capacity.

**Materials and methods:**

A case control study samples were collected of asthenozoospermic (n = 63) and normospermic (n = 43) cases, with average men age 30.35 ± 7.03 years old. Semen plasma specimens representing both fertile and sub-fertile men visiting two different secondary care health institute in Irbid province, Jordan. All samples were assessed according to WHO guidelines (2021) and by using spectrophotometry to evaluate the semen plasma levels of vitamin B_6_, glutathione (GSH) and total antioxidant capacity (TAC).

**Results:**

Our main finding is there is significant positive correlations between the seminal plasma concentration of GSH (p < 0.0001) and TAC (p < 0.0073) are significantly correlated with vitamin B_6_ deficiency in asthenozoospermia group in comparison to normozoospermia cases**.** A significant decrease (p < 0.0001) the levels of vitamin B6 in men with asthenozoospermia compared to normozoospermic men (control) with an approximate 80 % percent reduction in the mean levels between groups.

**Conclusions:**

These findings suggest that pyridoxine deficiency may very well alter the GSH system, in so doing affecting the antioxidant defense mechanism against reactive oxygen species to sperm, impacting sperm development and maturation. leading to asthenozoospermia.

## 1 Introduction

The imbalance between reactive oxygen species (ROS) production and antioxidant capacity leads to oxidative stress. Increased ROS production, a compromised antioxidant system, or a combination of the two is the cause to blame for this. The ROS attack will alter and denature functional and structural molecules in the presence of oxidative stress, resulting in tissue damage and malfunction [1]. It has been linked to numerous diseases including infertility. Male factor infertility contributes to nearly 50 % of all infertility issues, where it has been a medical concern due to the global decline in couples' capacity to reproduce. Sperm dysfunction has been linked to varicocele, hormonal imbalance, testicular cancer, urogenital tract infections, systemic diseases and genetic factors [2].

Recent evidence is mounting that oxidative stress may have a role in many aspects of male infertility [3,4]. A variety of enzymatic and nonenzymatic antioxidants regulate the biological oxidative effects of free radicals on macromolecules, while glutathione (GSH) is one example [5]. In seminal plasma, enzymatic antioxidants, such as glutathione peroxidase, and several non-enzymatic antioxidants (vitamin B_6_) that function as oxidant scavengers and cofactors of enzymatic antioxidants have been discovered. A growing evidence reveals vitamin B_6_ role in sperm maturation and sperm parameters and has been linked with semen quality [6]. Most importantly, vitamin B_6_ acts specifically as a coenzyme for cystathionine-β-synthase enzyme, which is essential for enabling the transsulphuration of homocysteine into cysteine amino acid [7]. Research studies have reported that a deficiency or a decrease in vitamin B_6_ concentration leads to hyperhomocysteinemia [8]. Given the fact that homocysteine is a toxic byproduct of methionine metabolism, its high concentration is found to be associated with the abnormal gonadal development and altered spermatogenesis in males [9]. Additionally, vitamin B_6_ itself has been well-documented as an antioxidant that provides protection of tissues against ROS [10]. In our recent study, it was concluded that normal concentration for vitamin B_6_ in semen should improve the antioxidant defense system and alleviate the progression of oxidative damage within sperm cells [11,12].

The male reproductive tract's oxidative environment is controlled by many systems, the glutathione being one of them which also aids in the proper development, maturity, and function of mammalian spermatozoa and acts as primary defense against excessive generation of harmful ROS [13]. The total antioxidant capacity (TAC) has been established as a diagnostic test that can be utilised in the male infertility workup [1]. TAC measures the amount of total antioxidants in seminal plasma. Therefore, it provides an assessment of the reductive potential in seminal plasma. Several studies have investigated the diagnostic application of TAC in various andrology conditions and numerous studies show that infertile patients have lower seminal TAC in comparison with fertile men [14]. Moreover, there is a positive correlation between reduced TAC and several seminal parameters, such as oligozoospermic and asthenozoospermic men comparison to normospermic [15].

Thus, in the current study, we investigated the seminal plasma GSH and TAC concentrations in ejaculated semen samples exhibiting abnormal sperm motility compared to samples with normal sperm parameters. Additionally, we explored the correlations between progressive and total motility, sperm concentration, semen volume, and the measured seminal plasma GSH and TAC within each tested group. To test the hypothesis that low seminal plasma vitamin B_6_ might adversely affect the sperm motility by altering the TAC and GSH in seminal plasma, we investigated the correlation between vitamin B_6_ and TAC/GSH in both tested groups.

## 2 Materials and methods

### 2.1 Study population

Between February 2018 and January 2019, both fertile and subfertile men visiting the Assisted Reproductive Technologies unit at King Abdullah University Hospital and Al-Qudah laboratories in Irbid province, Jordan, were included in this prospective study. In the present analysis, a total of one hundred and six male participants were evaluated. The study protocol and methodology were approved by the Institutional Review Board Committee of King Abdullah University Hospital-Jordan University of Science and Technology, Irbid, Jordan (193-2018). Prior to sample collection, signed informed consents were obtained from each participant.

All participants completed a comprehensive questionnaire, from which we extracted specific data points including medical history, selected body measurements (e.g.; height and weight), ethnicity, and lifestyle factors such as cigarette use and vitamin supplement intake. We excluded participants with a family history of infertility, reproductive issues such as orchidectomy, varicocele, or testicular tumors, chronic illnesses, and those currently using pharmaceutical drugs or vitamin supplements from this study. Also, participants with genetic causes of infertility and/or hormonal problems were excluded.

Participants were stratified in subgroups, including normozoospermia defined according to WHO 2021 by having a semen volume of 1.4 mL, total sperm count of ≥39 × 10^6^ cells/mL and progressive sperm motility of >30 %, whereas asthenozoospermia, were defined by having a progressive sperm motility of less than or equal to 30 %. Therefore, cases were divided into two groups: normozoospermic (control group, n = 43) and asthenozoospermic (n = 63). The average men age was 30.35 ± 7.03 years old of all 106 participants.

### 2.2 Semen collection and analysis

Semen specimens were collected via masturbation after a required abstinence period of 3–7 days. After liquefaction, the semen parameters of viscosity, volume, sperm concentration, sperm count, total and progressive motility, and percentage normal morphology were measured according to WHO 2021 guidelines [16]. Sperm motility and concentration were measured using a Makler counting chamber (Irvine Sci., Santa Ana, California, US) at a phase contrast optics of 200 × magnification. For this evaluation, a total of 10 μl from each semen sample were analyzed. Spermatozoa that were only linearly or circularly motile were regarded as progressive. For each replicate, 200 spermatozoa were evaluated in order to improve accuracy. All semen samples were centrifuged at 2500×*g* for 10 min and the supernatants seminal plasma were frozen without preservatives and then stored at −20 °C until assayed.

### 2.3 Analysis of vitamin B_6_ and oxidative stress biomarkers

#### 2.3.1 HPLC-MS/MS analysis of vitamin B_6_

To prepare the semen sample for LC-MS/MS analysis using high-performance liquid chromatography, a mixture was created by combining seminal plasma samples (250 μL) with 3 mL of chloroform and 250 μL of ethanol. This mixture was vigorously vortexed for 30 s. Subsequently, the samples underwent centrifugation at 2,500 g for 10 min to isolate proteins within the pellets. These precipitated proteins were reconstituted using 750 μL of methanol and then transferred into autosampler vials for HPLC-MS/MS analysis.

The seminal plasma vitamin B_6_ analysis was performed on 30AD SHIMADZU Binary HPLC system coupled with an 8,030 ESI-MS/MS system. Briefly, the chromatographic separation was primarily conducted on a revered phase HPLC column - Zorbax Eclipse Plus C18, 30 × 2.1 mm with 1.8 μm of particle size. The mobile phase was 2 % CH_3_OH, 4.8 g/L (NH₄)₂CO₃, and 98 % H_2_O. The injection volume was 10 μL, and the analytes were separated isocratically at a flow rate of 0.4 mL/min. The quantitative analysis took place on a tandem mass spectrometer (MS/MS) under specific conditions including a gas temperature of 200 °C, sheet gas flow at 11 L/min, overall gas flow at 10 L/min, sheet gas heater set to 350 °C, capillary voltage at 3500 V, charging voltage at 50 V, and nebulizer pressure of 40 psi. All measurements were conducted in duplicate using positive mode electrospray ionization (+ESI) with a sensitivity of 0.01 μg/L.

#### 2.3.2 Measurement of total glutathione concentrations in seminal plasma

Total glutathione (GSH) content within seminal plasma samples was measured using ThermoFisher Glutathione Colorimetric Detection Kit (EIAGSHC). In the presence of NADPH, oxidized glutathione is converted into reduced glutathione by glutathione reductase enzyme. The thiol group of GSH reacts with the chromogen to generate a colored compound, to be detected and measured at a wavelength of 405 nm. The glutathione standard curve was used to calculate the unknown total glutathione content in samples, which is directly proportional to the rate of chromophore production. To eliminate the interfering enzymes or proteins, the metaphosphoric acid is used [17].

#### 2.3.3 Measurement of total antioxidant capacity

Total Antioxidant Capacity (TAC) was assayed using Total Anti-Oxidative Capability Assay Kit (A015-2). The assay is basically dependent on the reduction of Cu^+2^ to Cu^+1^ by antioxidants. After the reduction reaction, Cu^+1^ ions react with a chromophore provided in the kit that generates a colored compound. This color is measured at a maximum absorbance of 490 nm. Uric acid standard curve was used to determine the concentration of antioxidant content in the samples [4].

### 2.4 Statistical analysis

GraphPad Prism 5.01 Computer Software (GraphPad Software Inc.) was used for carrying out the statistical tests. Kolmogorov-Smirnov test was used to test for normality of the dataset. Two-sided *t-*test was used to analyze the differences in seminal plasma GSH and TAC concentrations between men with asthenozoospermia and men with normozoopermia. Relationships between seminal plasma GSH and TAC levels, total and progressive motility, sperm count, semen volume, men age, and seminal plasma vitamin B_6_ concentrations was computed using Pearson's correlation coefficient. At a p-value of 0.05, the results were accepted as statistically significant. The data is reported as means ± standard deviations.

## 3 Results

The current study aimed to investigate the concentration of seminal plasma GSH and TAC in ejaculated semen samples with abnormal sperm motility compared to samples with normal sperm parameters and its correlations to progressive and total motility, sperm concentration, semen volume, and seminal plasma vitamin B_6_ in both tested groups. Table 1 revealed a significant decrease (p < 0.0001) the levels of vitamin B_6_ in men with asthenozoospermia compared to normozoospermic men (control) with an approximate 80 % percent reduction in the mean levels between groups. The alcohol consumption level among participants was zero due to Islamic religion beliefs and practices.

**Table 1:** The level of TAC, GSH, vitamin B_6_ and measured sperm parameters within normozoospermia and asthenozoopermia cases. Data are presented as means ± standard deviations and the range of each parameter.

Parameter	Normozoospermia (n = 43)		Asthenozoospermia (n = 63)		*P*-value
	Mean	range	Mean	range	
Age (years)	31.2 ± 7.2	20–45	29.7 ± 6.9	19–47	0.288
Smoker	Yes, n = 18	41.9 %	Yes, n = 24	38.1 %	0.175
Smoking length (years)	8.5 ± 4.5	1–10	8.04 ± 3.15	2–20	0.207
Semen volume (mL)	3.89 ± 1.8	1.4–8	3.02 ± 1.3	1.3–7	**0.0037**
Sperm concentration (Million/mL)	56.07 ± 19.1	20–105	24.92 ± 12.9	1–58	**<0.0001**
Progressive motility (%)	49.07 ± 9.0	35–65	18.17 ± 7.1	∼0.1–30	**<0.0001**
Total motility (%)	70.23 ± 11.5	50–100	35.48 ± 10.5	∼0.1–55	**<0.0001**
Vitamin B_6_ levels (μg/L)	3.7 ± 1.0	1.23–5.3	0.75 ± 0.4	0.95–1.48	**<0.0001**
TAC levels (mmol/L)	0.63 ± 0.01	0.16–0.80.	0.62 ± 0.08	0.21–0.76	0.909^a^
GSH levels (mmol/L)	12.47 ± 6.1	3.26–32.29	10.92 ± 5.2	3.79–27.44	0.164

a F-test comparing variances for TAC levels between the two groups showed significant p-value of 0.027. Highlighted p-values are significant for two-sided *t-*test.

**Supplementary Fig. 1:**
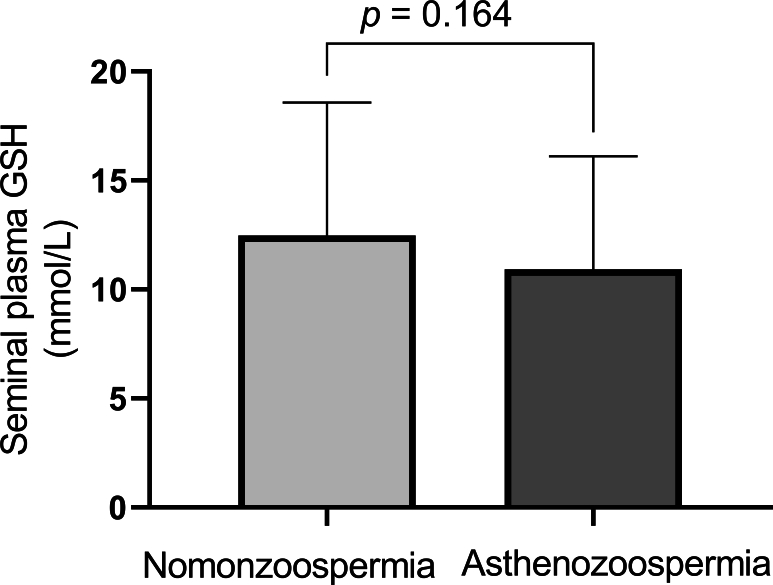
Concentrations of seminal plasma GSH in men with asthenozoospermia (n = 63) compared to normozoospermia (n = 43).

Supplementary figure 1 illustrated the concentrations of GSH in seminal plasma of men diagnosed with asthenozoospermia (n = 63) in comparison to men with normal sperm motility (control, n = 43), as determined by spectrophotometric analysis. The figure showed that there was no statistical significant difference (p = 0.164) observed in the GSH concentrations between the asthenozoospermic group and the control group.

**Supplementary Fig. 2:**
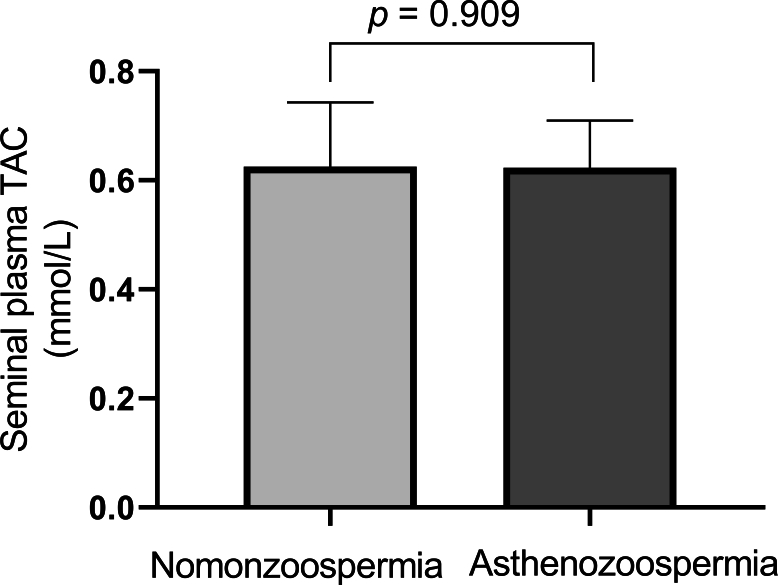
Concentrations of seminal plasma TAC in asthenozoospermia cases (n = 63) compared to normozoospermia (n = 43).

In Supplementary figure 2, the concentrations of TAC in seminal plasma are presented for both tested groups: men diagnosed with asthenozoospermia (n = 63) and control group (n = 43). These concentrations were also measured through spectrophotometric analysis. The figure revealed that there was no statistical significant difference (p = 0.909) detected in the TAC concentrations between the group with asthenozoospermia and the control group.

**Fig. 1:**
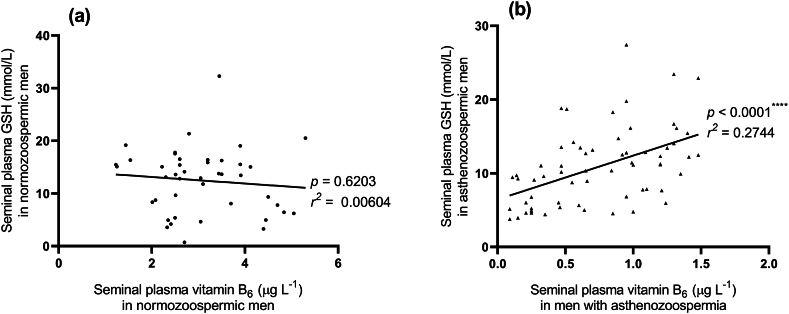
Pearson's correlation coefficient between seminal plasma GSH vs seminal plasma vitamin B_6_ in cases of normozoospermia (a, n = 43) and asthenozoospremia (b, n = 63).

The correlation between seminal plasma GSH and seminal plasma vitamin B6 in asthenozoospermic group (b, n = 63) compared to control group (a, n = 43) is depicted in Fig. 1. As seen in the figure, there is a statistical significant positive correlation between seminal plasma GSH and seminal plasma vitamin B6 in the asthenozoospermic group (p < 0.0001, r = 0.5238). However, no significant correlation was observed in the control group (p = 0.6203, r = −0.07774).

**Fig. 2:**
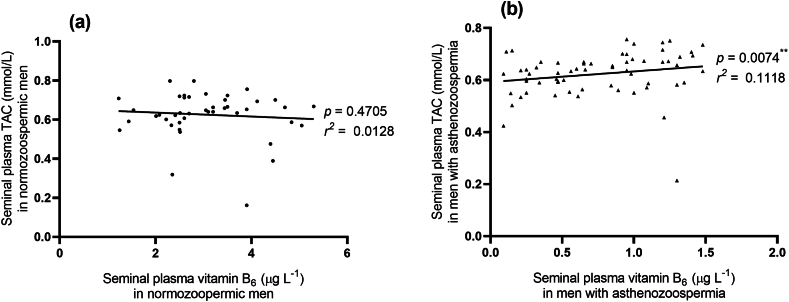
Pearson's correlation coefficient between seminal plasma TAC vs seminal plasma vitamin B_6_ in cases of normozoospermia (a, n = 43) and asthenozoospremia (b, n = 63).

Fig. 2 presents the correlation analysis between seminal plasma TAC and seminal plasma vitamin B6 in the asthenozoospermic group (b, n = 63) compared to the control group (a, n = 43). The figure illustrates a statistically significant positive correlation between seminal plasma TAC and seminal plasma vitamin B6 in the asthenozoospermic group (p = 0.0073, r = 0.3344). Conversely, no significant correlation was observed in the control group (p = 0.4705, r = 0.1130).

Table 2, Table 3 provide a comprehensive summary of the statistical correlation analysis conducted between various factors, including total motility of spermatozoa, progressive motility of spermatozoa, total sperm count, total semen volume, men's age, and seminal plasma vitamin B6, with seminal plasma GSH and TAC in both men with normal sperm motility and asthenozoospermic men.

**Table 2:** Summary of Pearson's correlation coefficient analysis of GSH in seminal plasma versus semen parameters (progressive motility, total motility, sperm concentration, semen volume), men age, and seminal plasma vitamin B_6_ in normozoospermia and asthenozoopermia cases).

Parameter	Normozoospermia (n = 43)			Asthenozoospermia (n = 63)		
	*p*-Value	r^2^	Correlation	*p*-Value	r^2^	Correlation
Total motility of spermatozoa	0.7775	0.00197	NS	0.1379	0.03572	NS
Progressive motility of spermatozoa	0.62	0.00604	NS	0.2072	0.02595	NS
Total sperm count	0.3091	0.02521	NS	0.8986	0.00026	NS
Total semen volume	0.5456	0.00897	NS	0.7219	0.00209	NS
Men age (19–47 years)	**0.0366**	0.10220	Sig.	0.4273	0.01036	NS
Seminal levels of vitamin B_6_	0.5074	0.01079	NS	**<0.0001**	0.23386	Sig.

**Table 3:** Summary of Pearson's correlation coefficient analysis of TAC in seminal plasma versus semen parameters (progressive motility, total motility, sperm concentration, semen volume), men age, and seminal plasma vitamin B_6_ in normozoospermia and asthenozoopermia cases).

Parameter	Normozoospermia (n = 43)			Asthenozoospermia (n = 63)		
	*p*-Value	r^2^	Correlation	*p*-Value	r^2^	Correlation
Total motility of spermatozoa	0.5812	0.007485	NS	0.897	0.000276	NS
Progressive motility of spermatozoa	0.6604	0.004755	NS	0.7913	0.001156	NS
Total sperm count	0.6253	0.005869	NS	**0.0551**	0.058951	NS
Total semen volume	0.8383	0.001027	NS	0.7105	0.002275	NS
Men age (19–47 years)	0.1063	0.062350	NS	**0.049**	0.062050	Sig.
Seminal levels of vitamin B_6_	0.5793	0.007560	NS	**0.0074**	0.111820	Sig.

## 4 Discussion

Infertility evaluation consisting of a reproductive history and at least one, preferably two, semen analysis is the recommended protocol. Interpreting semen analysis has been updated by WHO 2021, which consequently results in discrepancies where samples that would have been assessed abnormal if utilizing the 1999 handbook were instead considered to be normal [18]. Subsequently, the accuracy of routine semen analysis in assessing male fertility potential or predicting reproductive success is limited. Numerous studies revealed that using a mixture of sperm metrics to predict a man's reproductive status is crucial, as single sperm parameters have limited clinical significance for separating men who are fertile from men who have subfertility [19].

Oxidative stress and ROS have been under great attention lately to study male factor fertility, especially in idiopathic infertility. For example, Agarwal et al. suggest whether these patients have normal or abnormal semen parameters, ROS is an independent biomarker of male factor infertility [20]. The potential role of antioxidants was initially investigated on how GSH and superoxide dismutase affected the rate of acrosome response and loss of motility in centrifuged spermatozoa [21]. Several other studies also showed a decreased GSH level in the seminal plasma of infertile and sub-fertile groups compared with healthy fertile group [22,23]. In the current study, a trend of p-value 0.09 is observed in the GSH concentrations between the asthenozoospermic group and the control group, where our small sample size might be the culprit here. Moreover, no correlation was found between GSH nor TAC concentrations and the presence of specific sperm morphological defects, which further supports the potential use of GSH as a biochemical marker of idiopathic infertility [5].

One of the main findings emerging from this study is the correlation between seminal GSH, TAC and vitamin B6 from donors with asthenozoospremia. The glutathione system, including glutathione and its related enzymes, was found to function in neutralizing free radicals and protect sperm against oxidative injury [24]. A study on the intracellular sperm GSH system revealed samples from infertile men showed an alternation in the expression level of both GSH and the system's related enzymes in comparison it fertile samples [5]. The spermatozoa have its own oxidative defense enzymes including superoxide dismutase, glutathione peroxidases and glutathione reductase [25]. As such, glutathione peroxidases deficiency or absence has been associated with male infertility [26].

It is well established that the effects of vitamin B6 deficiency in the blood's glutathione level leading to a reduction in the glutathione/oxidized glutathione ratio [27]. Based on this knowledge and the evidence found in our study, vitamin B6 deficiency may very well alter the glutathione system by affecting the antioxidant defense mechanism against ROS to sperm, and impacting sperm development and maturation. Vitamin B6 is known for its strong antioxidant activity [28], serving as a coenzyme in the antioxidant defense system of GSH. The active form of vitamin B6, pyridoxal 5′-phosphate, serves as a coenzyme in the transsulfuration pathway of homocysteine to cysteine, which is a critical step contributing to the syntheses of reduced glutathione. Other than having a homocysteine lowering effect, it has been shown that treatment with supplementation results in reducing oxidative stress and ROS [29]. This incriminates to its immediate antioxidant activity. Our findings show the correlation between vitamin B6, GSH, TAC and sperm motility. In particular, human sperm with asthenozoospremia were found to have an increased levels of oxidative stress [30], while among healthy men, the lower motility of spermatozoa is associated with an increased oxidative stress in seminal plasma [31].

Therefore, given that higher levels of free radicals, particularly reactive oxygen species, in semen lead to oxidative stress, and sperm injury [[25], [31], [32], [33]], vitamin B6, once normally present in semen, may enhance the molecular defense mechanism against oxidative damage to sperm, thereby protects the normal sperm physiology, particularly sperm motility. Similarly, a case-control study in Iran found that adherence to the pattern comprising mainly of antioxidant nutrients may be inversely associated with asthenozoospermia [34]. Though, further research studies are still needed to fully understand the complex mechanism of oxidative stress management. Moreover, it is worthwhile to investigate the connection with the cysteine cycle as a key cofactor for cystathionine β-synthase is Vitamin B6. Limitations in our study include the several issues: we had limited budget, but we plan to further examine whether homocysteine concentration change is linked to abnormal semen parameters or infertility. Also, the participants were recruited from a specific population, which may limit the generalizability of the findings; thus, a larger sample size and more diverse populations are needed to validate and fully understand the underlying causes of male infertility. Finally, the determination of oxidation-reduction potential using TAC present important limitation due to sperm viscosity variation as well as no evidence-based reference is available due to the variation between machines and methodologies in detecting oxidizing capacity.

In conclusion, our study demonstrated that seminal plasma concentrations of GSH and TAC are significantly correlated with vitamin B6 deficiency in men with asthenozoospermia. These findings suggested that GSH concentration are recommended to be regularly checked in samples with impaired sperm motility in men with asthenozoospermia, providing valuable information by linking male infertility, seminal level of vitamin B6, and its effects on glutathione and total antioxidant capacity.

The findings of the current study confirmed the beneficial effect of vitamin B6 on sperm quality and maturation and advocate their use in the care and treatment of male infertility. However, further research studies are needed to better understand the underlying mechanisms and explore the potential clinical applications of glutathione as a diagnostic or therapeutic target for male infertility. However, data were collected from a single center representing only one geographical region in Jordan.

## Ethics statement

The study protocols and analysis was approved by the Institutional Review Board (IRB) Committee of King Abdullah University Hospital-Jordan University of Science and Technology, Irbid, Jordan (193-2018). Prior to sample collection, signed informed consents were obtained from every male recruit.

## Funding

This work was supported by Deanship of Research at Jadara University, grant number 778/13/112.

## Data availability statement

The data underlying this article are available in the article and in its online supplementary material.

## CRediT authorship contribution statement

**Roba Bdeir:** Writing – original draft, Project administration, Methodology, Funding acquisition, Formal analysis, Conceptualization. **Shefa’ Muneer Aljabali:** Writing – original draft, Data curation, Conceptualization. **Saleem Ali Banihani:** Writing – review & editing, Resources.

## Declaration of competing interest

The authors declare that they have no known competing financial interests or personal relationships that could have appeared to influence the work reported in this paper.

## Appendix A Supplementary data

Multimedia component 1 Multimedia component 1

Supplementary Table 1 Raw data of seminal analysis, vit B6, homocysteine, TAC and GSH concentrations among all samples. Supplementary Table 1
