# Supplementary material for: Role of pyridoxine and oxidative stress in asthenozoospermia
Source: Heliyon. 2024 Jul 19;10(14):e34799. doi: 10.1016/j.heliyon.2024.e34799 (PMC11325350; doi:10.1016/j.heliyon.2024.e34799)
Supplement: Multimedia component 1 [file mmc1.pdf]

جامعة العلوم والتكنولوجيا الأردنية

نموذج استبيان\ البحث العلمي

الاسم: ....

العمر: ....

الوزن: ....   الطول: ....

هل تعاني من أي مرض: (لا)   (نعم)   ما هو المرض: ....

هل تتناول أي أدوية: (لا)   (نعم)   ما هي الأدوية: ....

هل يوجد أحد في العائلة يواجه صعوبة في الإنجاب: (لا)   (نعم)

هل أنت مدخن: (لا)   (نعم) مدة التدخين: ....

هل تتناول مكملات غذائية (فيتامينات ب):   (لا)   (نعم)

 © All Rights Reserved

English translated version:

Name:
Age:
Weight:
Height:

Do you suffer from any disease?
No
Yes, specify ……………….

Are you taking any medications?
No
Yes, specify ……………….

Is there anyone in your family who is having difficulty conceiving?
No
Yes

Are you a smoker?
No
Yes, how long………….

Do you take nutritional supplements (like B vitamins)?
No
Yes
